# Coming together to improve access to medicines: The genesis of the East African Community’s Medicines Regulatory Harmonization initiative

**DOI:** 10.1371/journal.pmed.1003133

**Published:** 2020-08-12

**Authors:** Hiiti Sillo, Aggrey Ambali, Samvel Azatyan, Chimwemwe Chamdimba, Eliangiringa Kaale, Joseph Kabatende, Murray Lumpkin, Jane H. Mashingia, David Mukanga, Bonaventure Nyabenda, Gordon Sematiko, Margareth Sigonda, Burhani Simai, Fred Siyoi, Stanley Sonoiya, Mike Ward, Vincent Ahonkhai

**Affiliations:** 1 World Health Organization, Geneva, Switzerland; 2 African Union Development Agency–New Partnership for Africa’s Development, Midrand, South Africa; 3 School of Pharmacy, Muhimbili University of Health and Allied Sciences, Dar es Salaam, Tanzania; 4 Rwanda Food and Drugs Authority, Kigali, Rwanda; 5 Bill & Melinda Gates Foundation, Seattle, Washington, United States of America; 6 East African Community Secretariat, Arusha, Tanzania; 7 Directorate of Pharmacy, Medicines, and Laboratories, Bujumbura, Burundi; 8 National Drug Authority, Kampala, Uganda; 9 Zanzibar Food and Drug Agency, Zanzibar City, Zanzibar; 10 Pharmacy & Poisons Board, Nairobi, Kenya; 11 Gwynedd Consultancy, LLC, Philadelphia, Pennsylvania, United States of America

## Abstract

Hiiti Sillo and colleagues reveal how the East African Community’s Medicines Regulatory Harmonization initiative improves access to important medicines in Africa.

**Summary points**

- Independent, science-based regulation of medical products is a critical part of ensuring quality healthcare. When conducted in a transparent, science-based, efficient, accountable, and predictable manner, it can help ensure access to quality products that patients need.
- Several factors determine access to medicines, including treatment policy, pricing, and procurement, along with regulatory activities. Delays in regulatory filing and registration contribute to delays or lack of access to essential medicines in many African countries.
- We describe the solution to this problem developed by the East African Community (EAC) and launched in 2012: the EAC Medicines Regulatory Harmonization (MRH) initiative.
- Through the MRH initiative, the EAC hoped to increase the number of quality medicines that it registered by simplifying the application process for manufacturers. It also aimed to increase the speed at which it reviewed applications without decreasing rigor, by modernizing assessment processes and procedures.
- The EAC MRH initiative is innovative in many ways, particularly in its decentralized structure, emphasis on work-sharing, and regulation through reliance driven by trust and goodwill.

## Introduction

When tenofovir disoproxil fumarate (TDF) was approved by the United States Food and Drug Administration (FDA) in October 2001, it soon became an important treatment for HIV. It was the first novel antiviral agent approved in 6 years [1], and thanks to its favorable safety profile (it was associated with fewer and less frequent adverse events compared to the other antiretroviral drugs available at the time), it was especially important for individuals who could not tolerate the toxicity of the regimens then in current use [2]. Although TDF quickly became a treatment mainstay in the US and Europe, in early 2006 Médecins sans Frontières reported that the medicine’s manufacturer had only registered it for use in 5 sub-Saharan African countries: Gambia, Kenya, Rwanda, Uganda, and Zambia [3]. Even in the relatively large market of South Africa, the manufacturer did not apply for registration until late 2005 [3]. Streamlined regulatory systems can help encourage manufacturers to register their medicines in more countries and can minimize delays in approval. Independent, science-based regulation of medical products is a critical part of ensuring quality healthcare and, when conducted in a transparent, science-based, efficient, accountable, and predictable manner, it can help ensure access to quality products that patients need and can have a very positive impact on public health. When, however, regulation is not conducted in this manner, it can be a hindrance to such access and have a negative impact on public health. Of course, product registration/approval is only one step in a patient being able to access a needed quality medication; several important factors determine access to medicines, including treatment policy, pricing, and procurement, along with regulatory submission and approvals. However, delays in regulatory filing and registration contributed to TDF being “virtually unavailable” in many African countries for years [4]. Unfortunately, the delay between TDF’s approval in the US and its availability in many African countries is not unusual. An average of 4 to 7 years elapses between the first regulatory submission for a medicine—usually in a high-income country such as the US—and its approval in sub-Saharan African countries [5].

A number of factors, including policy recommendations and financing, play an important role in determining access to medicines in sub-Saharan Africa. However, a major driver of delays is the reluctance of medicines manufacturers to spend the time, effort, and expense necessary to register their products in each of Africa’s countries. To market their products, companies must submit lengthy applications to national medicines regulatory authorities (NMRAs), each of which typically has its own requirements and fees. Furthermore, the registration process often lacks transparency, with no clear timeline or accountability. In a 2012 survey, the majority of representatives from African pharmaceutical companies indicated that technical issues related to registration were problematic [6]. Paired with the fact that medicines manufacturers often view the potential profits for any one African country as relatively small, it is clear why the registration of new products has often been delayed, or even neglected entirely, in sub-Saharan Africa [7].

Regulators and policymakers throughout Africa have long recognized that improving the medicines registration process is essential for improving health outcomes. For example, African countries must contend with 75% of the world’s HIV/AIDS cases and 90% of its malaria deaths, and access to quality versions of the newest and most effective medicines is key in treating these infections [8], as well as in disease control strategies. Access to quality medicines is equally important for treating noncommunicable diseases, such as cardiovascular disease, diabetes, and cancer, which are predicted to overtake infectious diseases as the leading causes of death in Africa by 2030 [8]. To illustrate the power of improving regulatory efficiency, PATH recently estimated the effect of accelerating registration and scale-up for just 2 emerging medicines—one aimed at preventing postpartum hemorrhage and the other at treating pneumonia in children under the age of 5 years [9]. They found that speeding up time to patient access for these 2 medicines by just 2 years could save more than 23,000 lives in eastern and southern Africa. Optimizing the registration process has become increasingly important as Africa’s pharmaceutical market continues to grow, increasing in value from US$4.7 billion in 2003 to US$20.8 billion in 2013 and projected to reach US$40 billion to US$65 billion by 2020 [10].

In this article, we describe the solution to this problem developed by the East African Community (EAC), a regional economic community (REC) composed of Burundi, Kenya, Rwanda, South Sudan, Tanzania, and Uganda. In 2012, the EAC launched a region-wide Medicines Regulatory Harmonization (MRH) initiative, intended as a 5-year pilot for the broader African Union’s African Medicines Regulatory Harmonization (AMRH) initiative. In describing how the EAC MRH initiative moved from idea to reality, we hope to give other regions interested in instituting similar programs insight into important issues to consider, roadblocks likely to arise, and possible solutions for moving forward. We also hope that sharing the story of the initiative’s beginning will generate new interest in and ideas about addressing regulatory inefficiencies that hinder access to quality medicines in low- and middle-income countries.

### The genesis of the EAC’s MRH initiative

By the first decade of the new millennium, the need for technical harmonization and process optimization in regulating medicines had been recognized across the African continent. On the one hand, such harmonization and optimization would help incentivize medicines manufacturers to register their products in African countries, by decreasing the complexity of the application process [11]. On the other hand, harmonization could help NMRAs work more efficiently by allowing them to rely on their neighbors’ work when making their own regulatory decisions, thus minimizing duplicate assessments and inspections [11]. By freeing up time and resources in this way, NMRAs could process applications faster, speeding access to new products and focusing their resources on the public health issues that would deliver the most added value.

Two events during this period provided a foundation for action. In 2007, the Conference of African Ministers of Health and the African Union’s Heads of State and Government endorsed a Pharmaceutical Manufacturing Plan for Africa, which called for regulatory harmonization [8]. Then, in 2008, many African regulators met at the Thirteenth International Conference of Drug Regulatory Authorities, held in Bern, Switzerland, to discuss opportunities for cooperation. At this conference, the regulators requested that the World Health Organization (WHO) use its convening authority to bring countries together and use its technical norms and standards authority to drive the regulatory harmonization agenda forward. These WHO mandates are granted by the World Health Assembly and the International Conference of Drug Regulatory Authorities. Historically, many countries have relied on WHO for technical assistance in many fields, including regulatory capacity building, and requests for assistance are presented to WHO via both formal and informal WHO member state mechanisms, such as the request presented at this conference.

**Fig 1:**
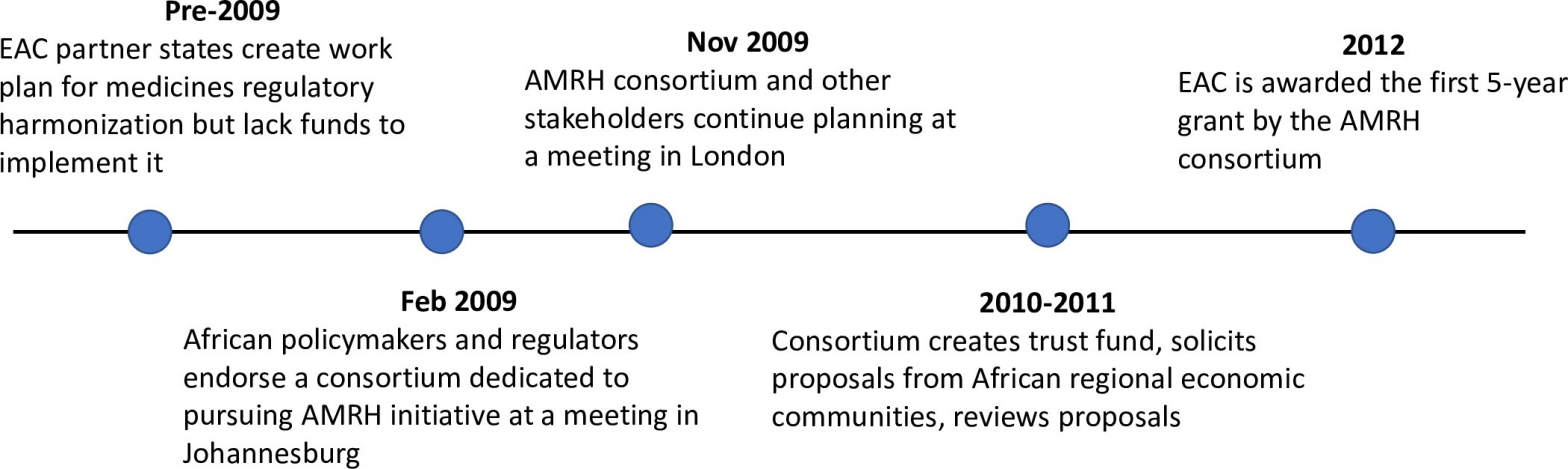
Timeline of major events leading to the creation of the EAC’s MRH initiative, a pilot project for the broader AMRH initiative. AMRH, African Medicines Regulatory Harmonization; EAC, East African Community; MRH, Medicines Regulatory Harmonization.

Planning for continent-wide MRH began in earnest in 2009 (Fig 1). In February of that year, the African Union Development Agency–New Partnership for Africa’s Development (AUDA-NEPAD) and the Pan-African Parliament (PAP) cohosted a meeting. There, policymakers and regulators from almost 40 African countries endorsed the objectives of a consortium dedicated to pursuing an AMRH initiative. The consortium included regulatory and political bodies (AUDA-NEPAD, PAP, the African Union Commission, the Heads of NMRAs, the Joint United Nations Programme on HIV/AIDS), the World Bank, technical partners (WHO, the Swiss Agency for Therapeutic Products [Swissmedic]), and donors (the Bill & Melinda Gates Foundation, the Clinton Health Access Initiative, the UK Department for International Development) [12]. Another meeting, hosted by AUDA-NEPAD and WHO, followed that November. There, the consortium began to finalize the details of its plan with input from other stakeholders, such as the Global Fund to Fight AIDS, Tuberculosis, and Malaria; the European Medicines Agency; and the International Federation of Pharmaceutical Manufacturers & Associations.

Though its immediate focus was on registering generic medicines, the consortium’s ultimate vision was to coordinate the entire landscape of medicines regulation, from clinical trials oversight to medicines registration to pharmacovigilance, across the continent. To pursue this vision, the consortium decided to work through Africa’s RECs [12]—groups of countries that work together through treaties to promote economic integration, including the free movement of goods. At this time, there was only sufficient funding for the consortium to support one pilot project. Therefore, the consortium decided to solicit proposals from each of the RECs and fund the most promising plan for MRH over the next 5 years. Together, WHO and AUDA-NEPAD established requirements for the proposals, as well as evaluation criteria. These criteria included how meaningful the proposed activities would be, in terms of regulatory harmonization; how well the objectives were described; and how much a given REC had already demonstrated that its partner states could cooperate to achieve common goals.

In 2012, the EAC, which at that time accounted for 14% of Africa’s population (and did not yet include South Sudan), was chosen as the REC that would pilot the initiative [11,13]. Unlike some RECs, the EAC consists of a small number of partner states, most of which share a common history, language, culture, and infrastructure, facilitating cooperation [7]. At the time of application, a customs union and common market were already in force in the EAC, monetary union was planned for 2024, and there were even future plans for a political federation in which the EAC would become a “super state” [14]. In addition, Chapter 21, Article 118 of the Treaty for the Establishment of the EAC, signed in 1999, provided a political platform for MRH across the region. It called for partner states to “harmonise drug registration procedures so as to achieve good control of pharmaceutical standards without impeding or obstructing the movement of pharmaceutical products within the Community,” as well as “develop a common drug policy which would include establishing quality control capacities and good procurement practices.” Finally, NMRA staff from Kenya, Tanzania, and Uganda had already been working closely with each other and with WHO staff during a pilot project in which medicines registration applications to NMRAs and WHO’s Prequalification Programme were assessed jointly [12].

Despite these advantages, the EAC faced a substantial challenge in building a regional regulatory system in one of the fastest growing regions in Africa. In 5 short years, this system would have to work for nearly 150 million people, spread across roughly 2 million square kilometers [7,15]. In addition, the system would have to contend with the fact that all partner states except for Kenya were low-income countries, as classified by the World Bank [7]. Partner states’ health systems were grappling with major problems: life expectancy across the region was below the global average, and the infant mortality rate was above the African average [11,13].

The partner states of the EAC were highly motivated to make the initiative a success. As early as 2000, the EAC Integrated Council of Ministers had asked a Research, Policy, and Health Systems Working Group to draft a common medicines policy, as well as harmonized medicines regulatory procedures [7]. In addition, in 2005, the NMRAs of Uganda, Tanzania, and Kenya had created a work plan for a region-wide MRH initiative. Unfortunately, their ability to implement the plan at that time was stymied by a lack of resources. Nevertheless, they continued to lay the groundwork for regulatory harmonization, and in 2010, AUDA-NEPAD engaged consultants to conduct an in-depth evaluation of the regulatory capacity and scope of activities being conducted by each partner state [7]. This prior planning made developing an MRH proposal straightforward, and the EAC partner states were eager to use the initial US$5.5 million award from the AMRH program to turn their long-held dream into a reality. The EAC MRH initiative was officially launched in March 2012 in Arusha, Tanzania [12].

### The goals of the EAC’s MRH initiative

**Fig 2:**
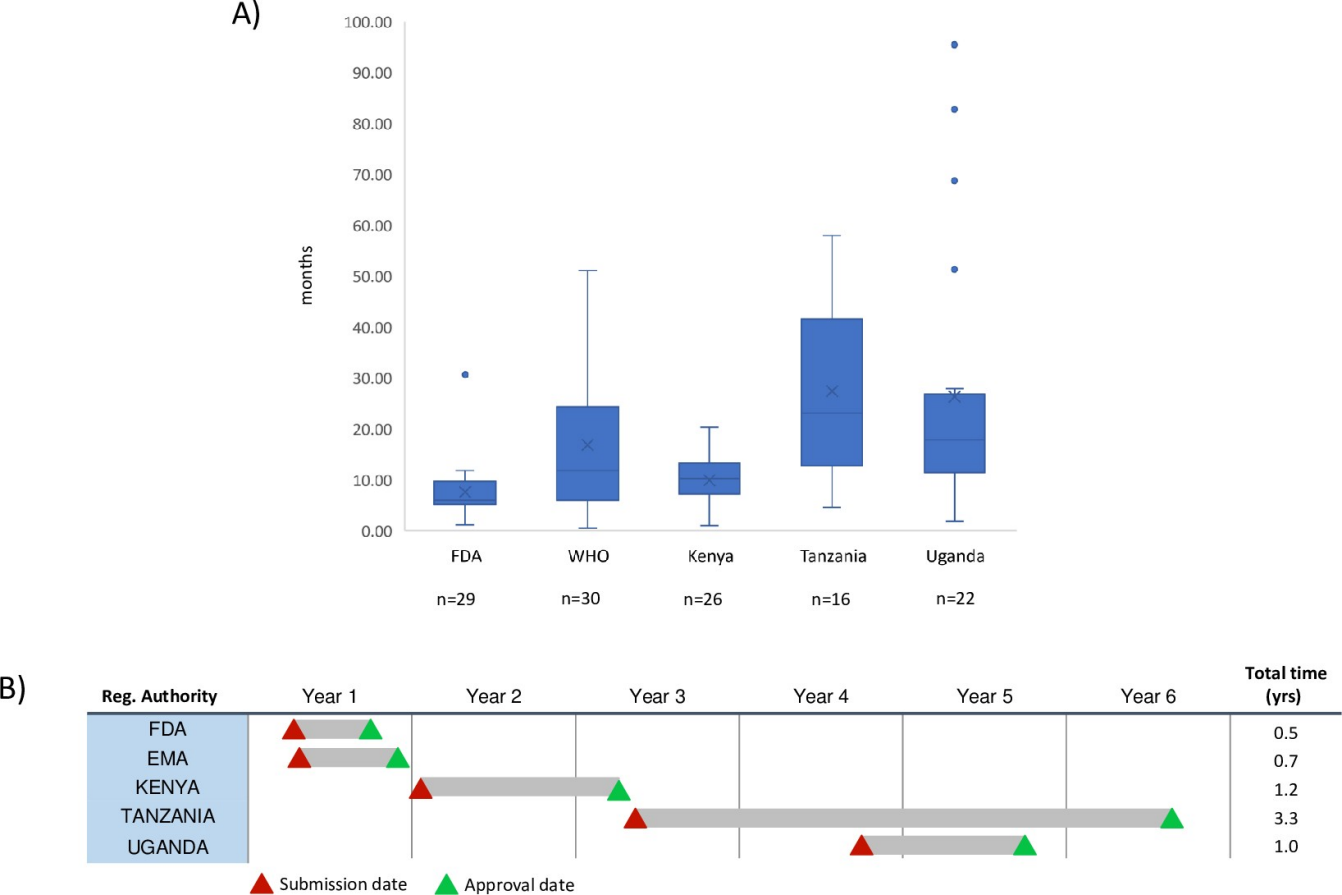
Time to approve marketing authorization applications in the EAC prior to the EAC MRH initiative. When the EAC’s MRH initiative began, there was substantial inter- and intra-agency variation in the length of time that NMRAs took to approve marketing authorization applications submitted by global pharmaceutical companies. (A) Median, minimum, and maximum times from the submission of a registration application until its approval by the NMRAs of Kenya, Tanzania, and Uganda. For comparison, times for the US FDA and WHO are shown as well. Data were provided by 10 global pharmaceutical companies to the Boston Consulting Group with the understanding that company and drug names would remain confidential and spanned medicines and vaccine submissions from 1985 to 2012. (B) Even for the same medicine, the time from submission to approval could vary dramatically between NMRAs. Here the time from submission to approval of a single antiretroviral medicine initially submitted to the FDA during the 1985 to 2012 time period is shown for the NMRAs of Kenya, Tanzania, and Uganda, as well as the FDA and EMA for comparison. *Source information for Figure from: Ahonkhai et al 2016 [5].* EAC, East African Community; EMA, European Medicines Agency; FDA, Food and Drug Administration; MRH, Medicines Regulatory Harmonization; NMRA, national medicines regulatory authority; WHO, World Health Organization.

Ultimately, through region-wide cooperation, the EAC hoped to increase the number of quality medicines it registered by simplifying the application process for manufacturers. It also aimed to increase the speed at which it reviewed applications without decreasing rigor, by modernizing assessment processes and procedures. This represented a challenge. Although most EAC partner states allowed medicines manufacturers to submit abridged dossiers for agents that had already been approved by a recognized regulatory authority, such as the US FDA or the European Medicines Agency, local approval processes still took an average of 24 months [7]. The reasons for these long regulatory timelines, which have been well described by Ahonkhai and colleagues [5], included redundant steps in the technical reviews, ineffiencies in the regulatory processes themselves, and manufacturers not meeting quality standards. Moreover, wide variation within and between partner states existed in the length of time taken to process applications (Fig 2), and there was often little transparency in the application process or clarity about processing timelines. Finally, there was little communication between EAC partner states about regulatory decisions.

To meet its overarching goal of improving access to quality medicines, the EAC MRH initiative decided to focus on

- Developing and implementing
  - A Common Technical Document that manufacturers could use to register medicines within any EAC partner state
  - A common information management system for medicines registration that would link all partner states, as well as the EAC Secretariat
  - A quality management system in each NMRA, to ensure that each partner state carried out regulatory activities in a uniform and rigorous manner
- Building the EAC’s regional and national capacity to implement registration processes and harmonize and align technical standards
- Developing and implementing a framework for partner states to eventually recognize the regulatory findings and decisions of their neighbors

The initiative decided that an early focus would be adapting the Common Technical Document format and guidelines created by the International Council for Harmonisation of Technical Requirements for Pharmaceuticals for Human Use, as well as resources from sources such as the WHO Medicines Prequalification Programme and the European Medicines Agency, for use in the EAC setting.

At the time the pilot grant was awarded, the regulatory infrastructure of the EAC’s partner states varied dramatically [15]. Whereas Kenya, mainland Tanzania, and Uganda had well-established NMRAs, Burundi, Rwanda, and Zanzibar had not yet established NMRAs [7]. (Zanzibar is a semi-autonomous region of the United Republic of Tanzania whose government is responsible for its own health affairs; therefore, it has its own NMRA. As shorthand, in this collection we refer to mainland Tanzania’s NMRA as Tanzania’s NMRA.) Furthermore [7],

- Each partner state that registered medicines was using a different format for its registration applications.
- Only the NMRAs of Kenya, Tanzania, and Uganda had guidelines for registering medicines in place.
- Only Kenya, Tanzania, and Uganda had the ability to assess new products (versus generics or products that had already been approved by a recognized regulatory authority).
- Only Kenya, Tanzania, and Uganda had national guidelines that met WHO standards for good manufacturing practice (GMP).
- In terms of quality management, only Tanzania’s NMRA had achieved International Organization for Standardization (ISO) 9001:2008 certification. This certification is the measure of an agency’s quality management system as judged by ISO standards. Meeting the requirements for ISO certification is a globally recognized accreditation of the maturity and functionality of the quality management system of an organization, including a medical products regulatory agency.
- Only Kenya, Tanzania, and Uganda had fully functioning electronic information systems that could be used to share data and track the progress of applications.
- EAC NMRAs were severely understaffed (Table 1). For example, Kenya and Uganda each had 11 qualified staff evaluators; Rwanda and Zanzibar each had only 2.

**Table 1:** Comparison of regulatory agency total staff numbers, as of 2013^a^.

	Burundi	Rwanda	Kenya	Tanzania	Uganda	UK	US
**Population (in millions)**	10	12	44	49	38	68	335
**Agency staff size**	26	11	200	270	149	900	~10,000
**Staff per million residents**	2.6	0.92	4.6	5.5	3.9	13.2	29.9
	Region average: 3.5 staff per 1,000,000 residents						

^a^These data were collected at the start of the EAC’s MRH initiative in 2012–2013. *Source*: *Bill & Melinda Gates Foundation*.

**Abbreviations:** EAC, East African Community; MRH, Medicines Regulatory Harmonization

As a result of these differences, the initiative had to incorporate a substantial amount of capacity building so that partner states would be able to trust their neighbors to process applications according to a shared standard and to produce quality data and documents [5,16,17].

### The structure of the EAC’s MRH initiative

The EAC designed its initiative’s structure with these goals in mind, also taking into account the existing framework of the EAC. Many existing regional bodies rely on a central authority to conduct regulatory functions, as well as a legal framework that binds partner states to the decisions of that central authority. It was not feasible for the EAC to create such a central authority during the 5 years of its pilot grant; rather, the goal was to implement a regional regulatory system that functioned well and could be established quickly. For that reason, the EAC pioneered a new, decentralized regulatory system in which different partner states took primary responsibility for different regulatory functions. This approach facilitated specialization while ensuring that all countries were fully engaged in the initiative. Although partner states harmonized technical requirements and performed joint assessments and inspections, final decisions regarding product authorization would continue to reside at the national level; work-sharing and reliance on the regulatory products of partner agencies would be driven by trust and goodwill, due to existing legal requirements. Partner states would also now implement regional regulatory activities, such as joint product assessments and joint GMP inspections, alongside their existing national regulatory processes.

**Fig 3:**
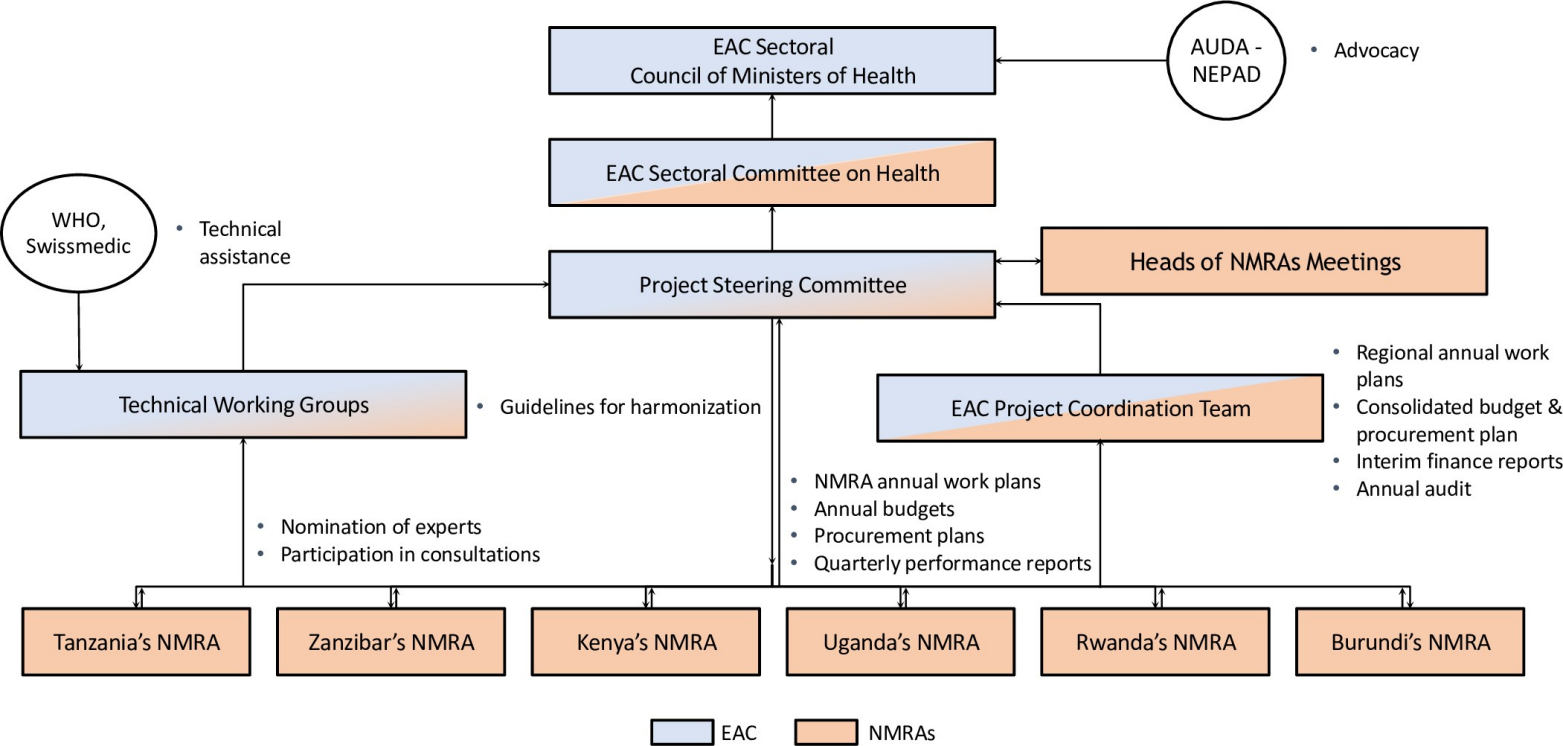
Governance structure of the EAC’s MRH initiative. AUDA-NEPAD, African Union Development Agency–New Partnership for Africa’s Development; EAC, East African Community; MRH, Medicines Regulatory Harmonization; NMRA, national medicines regulatory authority; Swissmedic, Swiss Agency for Therapeutic Products; WHO, World Health Organization.

The EAC MRH program relied on a Steering Committee, technical working groups, and a Project Coordination team to help carry out its functions (Fig 3). The Steering Committee consisted of the Heads of each partner state’s NMRA, the chief pharmacists of each partner state, the EAC Secretariat, and AMRH partners. This committee met twice a year to approve work plans and budgets, as well as review and endorse guidelines. The initiative also capitalized on a model already being used successfully by the EAC: the technical working group. On the basis of each NMRA’s strengths, the initiative assigned leadership roles:

- Tanzania, which possessed the most developed semiautonomous NMRA [15], would lead the Medicines Evaluation and Registration Working Group.
- Uganda would lead the GMP Inspections Working Group.
- Rwanda would lead the Information Management Systems Working Group.
- Kenya would lead the Quality Management Systems Working Group.

These working groups, which typically included 2 representatives from each partner state, as well as staff from the EAC Secretariat, AUDA-NEPAD, WHO, and development partners, met at least twice a year to draft technical guidelines and procedures, which were presented to the Steering Committee for review and endorsement. Finally, a Project Coordination team—which consisted of a project coordinator; a health and informatics officer; an accountant; a pharmaceutical program assistant; and 6 focal staff, one located in each NMRA—was responsible for overall project planning, preparation, procurement, execution, monitoring, evaluation, fund management, and reporting.

**Fig 4:**
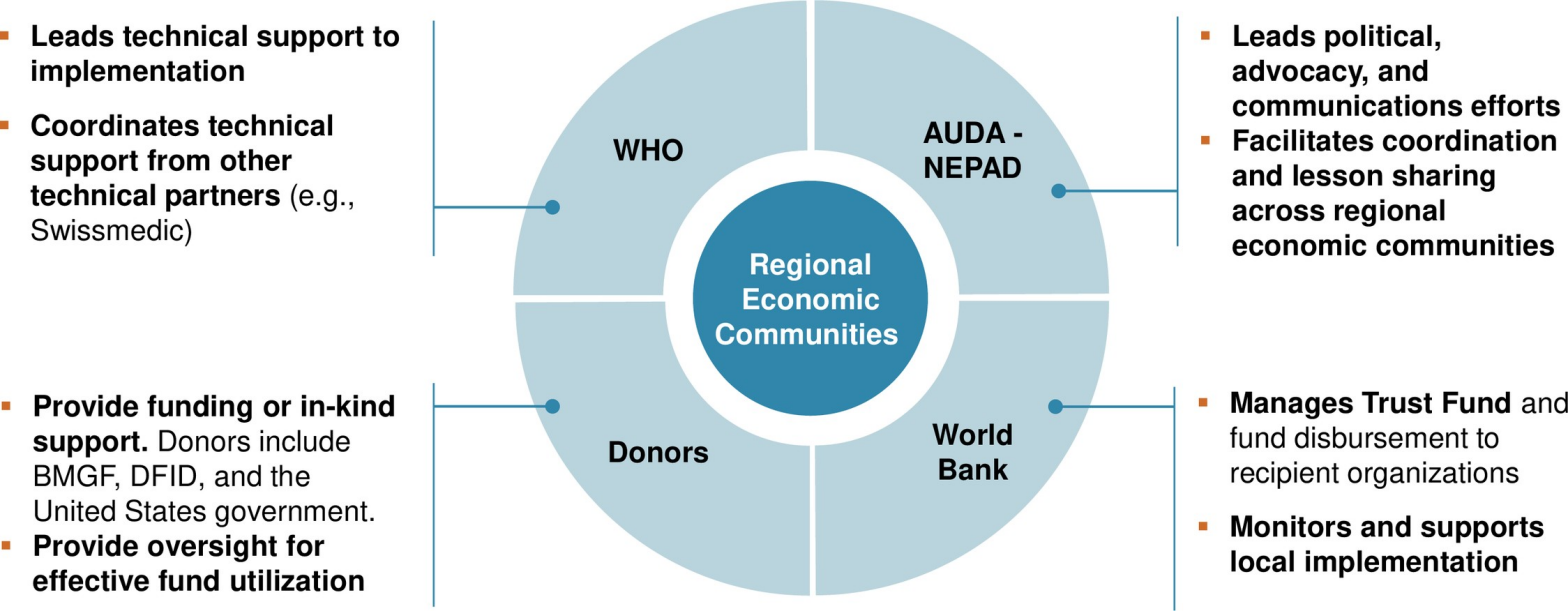
Role of partners in the EAC’s MRH initiative. AUDA-NEPAD, African Union Development Agency–New Partnership for Africa’s Development; BMGF, Bill & Melinda Gates Foundation; DFID, UK Department for International Development; EAC, East African Community; MRH, Medicines Regulatory Harmonization; Swissmedic, Swiss Agency for Therapeutic Products; WHO, World Health Organization.

The initiative also assigned important roles to external partners (Fig 4). The World Bank, for example, oversaw the initiative’s finances, administering funds from the AMRH multi-donor trust fund. The World Bank disbursed funds to the EAC Secretariat, which itself managed project funds for the EAC. WHO and Swissmedic both committed to providing technical support to the EAC Secretariat and partner states’ NMRAs, as well as training in how to meet current international standards for quality assessment and GMP inspections. Finally, AUDA-NEPAD helped coordinate the various stakeholders involved in the initiative.

To help build the capacity of partner states with less mature regulatory systems, the initiative designed a twinning system. Newly established regulatory systems were paired with more established NMRAs (Zanzibar’s NMRA was paired with Kenya’s, Burundi’s with Tanzania’s, and Rwanda’s with Uganda’s). By working together on joint activities, such as product assessments and GMP inspections, more mature NMRAs had the opportunity to pass along best practices, expertise, and institutional knowledge. In addition, this approach allowed the twinned NMRAs to build relationships and confidence so that staff felt comfortable communicating with one another outside of the joint activities. Relationships were also strengthened through staff exchanges; for example, staff from Rwanda’s NMRA would spend time in Uganda’s NMRA, and vice versa. These exchanges allowed NMRAs to learn from the operations and standard operating procedures of other NMRAs, including those with greater experience and expertise.

## Conclusion

Throughout the planning process for its MRH initiative, the EAC’s primary focus was facilitating access to quality medicines through harmonization of technical requirements, regulatory process optimization, and regional cooperation. The EAC was well aware of the initiative’s potential benefits for its own residents. However, it was also aware that the pilot’s results would be used to determine whether it was feasible for other African RECs to begin their own harmonization and optimization initiatives—and, ultimately, whether it made sense to pursue an initiative that would span the entire continent [11]. The EAC MRH initiative was innovative in many ways, particularly in its decentralized structure, emphasis on work-sharing, and regulation through reliance driven by trust and goodwill. In the next paper in this Collection, “Eight years of the East African Community Medicines Regulatory Harmonization initiative: Implementation, progress, and lessons learned” [18], we will describe what this innovative approach allowed the initiative to accomplish, including its most significant achievements and its biggest challenges.

## Abbreviations

AMRH: African Medicines Regulatory Harmonization
AUDA-NEPAD: African Union Development Agency–New Partnership for Africa’s Development
EAC: East African Community
FDA: United States Food and Drug Administration
GMP: good manufacturing practice
ISO: International Organization for Standardization
MRH: Medicines Regulatory Harmonization
NMRA: national medicines regulatory authority
PAP: Pan-African Parliament
REC: regional economic community
Swissmedic: Swiss Agency for Therapeutic Products
TDF: tenofovir disoproxil fumarate
WHO: World Health Organization
